# A multi-omics machine learning framework in predicting the recurrence and metastasis of patients with pancreatic adenocarcinoma

**DOI:** 10.3389/fmicb.2022.1032623

**Published:** 2022-11-03

**Authors:** Shenming Li, Min Yang, Lei Ji, Hua Fan

**Affiliations:** ^1^Department of Hepatobiliary and Pancreaticosplenic Surgery, Beijing Chaoyang Hospital, Capital Medical University, Beijing, China; ^2^Department of Nephrology, Essen University Hospital, University of Duisburg-Essen, Essen, Germany; ^3^School of Electrical and Information Engineering, Anhui University of Technology, Ma’anshan, Anhui, China; ^4^Genesis Beijing Co., Ltd., Beijing, China

**Keywords:** pancreatic adenocarcinoma, multi-omics, microbial community, random forest, local recurrence, distant metastasis

## Abstract

Local recurrence and distant metastasis are the main causes of death in patients with pancreatic adenocarcinoma (PDAC). Microbial content in PDAC metastasis is still not well-characterized. Here, the tissue microbiome was comprehensively compared between metastatic and non-metastatic PDAC patients. We found that the pancreatic tissue microbiome of metastatic patients was significantly different from that of non-metastatic patients. Further, 10 potential bacterial biomarkers (*Kurthia*, *Gulbenkiania*, *Acetobacterium* and *Planctomyces* etc.) were identified by differential analysis. Meanwhile, significant differences in expression patterns across multiple omics (lncRNA, miRNA, and mRNA) of PDAC patients were found. The highest accuracy was achieved when these 10 bacterial biomarkers were used as features to predict recurrence or metastasis in PDAC patients, with an AUC of 0.815. Finally, the recurrence and metastasis in PDAC patients were associated with reduced survival and this association was potentially driven by the 10 biomarkers we identified. Our studies highlight the association between the tissue microbiome and recurrence or metastasis of pancreatic adenocarcioma patients, as well as the survival of patients.

## Introduction

Pancreatic adenocarcinoma (PDAC) remains one of the most lethal malignancies, owing in part to its early onset of metastasis (Roe et al., 2017). Most PDAC patients have metastasized at the time of diagnosis, when there is minimal benefit from surgical or chemotherapy interventions (Ryan et al., 2014; Liu et al., 2021). Consequently, only 5% of PDAC patients survive more than 5 years after diagnosis because of its unpredictability (Chen, 2015). Improving the dismal prognosis requires a better understanding of the mechanisms of PDAC metastasis, especially the identification of metastasis biomarkers.

The microbiota inhabiting the human body is estimated to be between 10 and 100 trillion (Costello et al., 2009). While most microorganisms reside in the gastrointestinal tracts, microbiota can be found in other organs and tissues (Li et al., 2021). They play an important role in maintaining body homeostasis, and dysbiosis of the microbiota may contribute to the pathogenesis of many diseases (Liang et al., 2018). Growing researches have suggested that microbial communities influence the occurrence, progression, and response to therapy of pancreatic adenocarcioma and other cancers (Fan et al., 2018; Riquelme et al., 2019; Yang M. et al., 2022). For example, studies have shown that cancerous pancreas has significantly richer microbiota compared to normal pancreas (Pushalkar et al., 2018). Recently, Riquelme et al. (2019) found that interaction between pancreatic adenocarcioma microbiome composition and gut microbiome affects host immune responses. Besides, studies have shown that oral antibiotic depletion of gut microbiota in mice suppresses tumor growth and metastasis while activating antitumor immunity in the tumor environment (Wei et al., 2019; Liu J. et al., 2022). However, the potential association between microbial communities of cancer tissue and pancreatic adenocarcioma metastasis remains a knowledge gap.

The occurrence and development of pancreatic adenocarcioma are affected by multiple factors. Previous studies have revealed that the development of pancreatic adenocarcioma is accompanied by changes in the expression patterns of large set of mRNAs (He et al., 2022) and non-coding RNAs, such as lncRNAs and miRNAs (Xiao et al., 2018; Wang et al., 2019; Xu et al., 2020b; Zhang et al., 2020). LncRNA PSMB8-AS1 contributes to pancreatic adenocarcioma progression *via* modulating miR-382-3p/STAT1/PD-L1 axis (Zhang et al., 2020). LncRNA DANCR promotes proliferation and metastasis in pancreatic adenocarcioma by regulating miRNA-33b (Luo et al., 2020). Wang et al. (2020) reported that the upregulation of METTL14 led to the decrease of PERP levels *via* m^6^A modification, promoting the growth and metastasis of pancreatic adenocarcioma. Sohrabi et al. (2021) found that 6 out of 43 common miRNAs (hsa-miR-210, hsa-miR-375, hsa-miR-216a, hsa-miR-217, hsa-miR-216b, and hsa-miR-634) had significant differences in their expression profiles between the tumor and normal groups of pancreatic adenocarcioma. However, comparative studies on the accuracy of different omics in predicting recurrence and metastasis in pancreatic adenocarcioma patients are still vacant.

In this study, 37 samples of patients with recurrence or metastasis (RM) and 42 samples of patients without recurrence or metastasis (no-RM) were collected, and the tissue microbiome of all patients with pancreatic adenocarcioma were characterized. The main objectives of this study were: (1) to identify the bacterial biomarkers capable of discriminating between RM and non-RM, (2) to compare the differences in transcriptome levels between RM and no-RM patients, and (3) to compare the performance of microbes and mRNAs in predicting pancreatic adenocarcioma recurrence or metastasis. Our study sheds light on the ability of tissue microbial biomarkers of pancreatic adenocarcioma to predict recurrence or metastasis.

## Materials and methods

### Sampling populations and datasets

Microbiome data and transcriptome data were obtained from the Cancer Genome Atlas (TCGA) database.^1^ The microbiome data of pancreatic adenocarcioma patient tissues were derived from the re-cleaning of the sequencing data of samples from the TCGA database by Rob Knight’s team (Poore et al., 2020). The microbial RNA data of pancreatic adenocarcioma patients were selected and the clinical data of pancreatic adenocarcioma in TCGA were downloaded. The samples were divided into two groups according to whether the patients had recurrence or metastasis within 1 year after the initial diagnosis. Patients with recurrence or metastasis or both within 1 year were defined as RM, and those without recurrence or metastasis were defined as no-RM. In total, we matched 79 samples, including 37 RM and 42 no-RM. We also collected some essential clinical indicators of the patients, such as age, gender, and disease stage, etc.

### Statistical analysis

Statistical analysis was performed using R language. Wilcoxon rank sum test was used to determine the relationship between different clinical features and patients’ recurrence and metastasis. If the *p*-value between the two groups is less than 0.05, it is considered that there is a statistically significant difference. At the same time, by constraining the *p*-value to be less than 0.01, the microbial characteristics with significant differences were screened as potential microbial markers for downstream analysis.

### Identification of differentially expressed genes

Differentially expressed genes (DEGs) of mRNA, lncRNA, and miRNA were identified using the “Deseq2” R package. Up-regulated genes were obtained by adjusted *p*-value < 0.1 and log2 Fold Change > 0. Down-regulated genes were obtained by adjusted *p*-value < 0.1 and log2 Fold Change < 0. Then, genes with significant differences were screened by | log2 (Fold Change)| ≥ 1 and adjusted *p*-value less than 0.05. Significantly different genes were displayed by the “pheatmap” package in R. Gene Ontology (GO) enrichment analysis was conducted by the “clusterProfiler” package in R. Enrichment pathways of DEGs were displayed by the “ggplot2” package in R.

### Diversity analysis

Alpha-diversity (Richness, Chao, Shannon, and Simpson indices) were calculated using the “vegan” package in R. Principal coordinate analysis (PCoA) was conducted with the “vegan” package in R to analyze differences between microbial communities. Wilcoxon rank sum test was used for two group comparisons of microbial diversity. *P*-value less than 0.05 was considered statistically different.

### Machine learning classification model

To evaluate the performance of different omics in predicting the recurrence and metastasis of patients with PDAC, we labeled the RM patients as “0” and the no-RM patients as “1,” which turned our research into a binary classification of machine learning. Random Forest (RF) model in Python’s Sklearn module was used for classification. RF randomly samples all the original data, generates n different sample datasets, builds a decision tree model for each dataset, and finally obtains the prediction result of the final model according to the voting results of each decision tree model. We estimated the performance of the classification algorithms using the fivefold cross-validation (fivefold-cv). The performance of the classification algorithm was calculated by averaging the AUC (area under curve) in the five test datasets. Finally, metrics including AUC, ACC (accuracy), precision, recall, and F1-score were used to comprehensively evaluate the performance of the model.

### Survival prediction

Ten bacterial biomarkers were identified using Wilcoxon rank sun test. Then, these 10 biomarkers were used to predict the survival of patients with PDAC. The survival curve was conducted using the Kaplan–Meier (KM) method and log-rank test was used to compare the difference of survival probability. The analysis and visualization were conducted with the “survival” package in R.

## Results

### Tumor node metastasis classification stages are significantly correlated with recurrence and metastasis of pancreatic adenocarcioma

**FIGURE 1:**
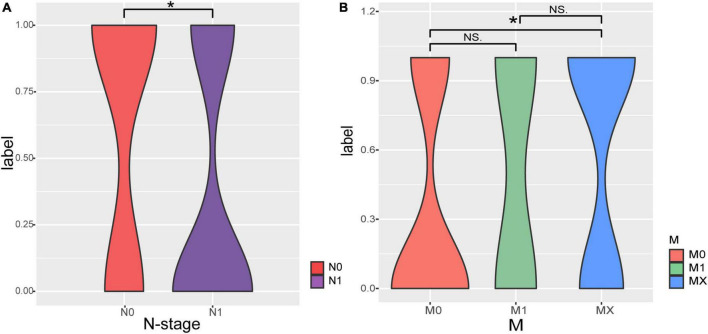
The correlations of Tumor Node Metastasis classification (TNM) stage with recurrence and metastasis. **(A)** Patients with recurrence or metastasis are accompanied by increased lymph node involvement. **(B)** Comparisons of M staging in patients with RM and no-RM; Wilcoxon test is used to compare between different groups of samples. The *X*-axis represents the different stages of patients; *Y*-axis represents the recurrence and metastasis, 0: recurrence or metastasis; 1: without recurrence and metastasis.

The correlations between clinical phenotype and recurrence and metastasis of pancreatic adenocarcioma patients were shown in Table 1. Both N stage and M stage have significant differences between RM and no-RM (Figure 1). Specifically, PDAC patients with advanced disease (N1) had a significantly increased probability of recurrence or metastasis. That means PDAC patients with recurrence or metastasis were accompanied by increased lymph node involvement. Besides, there were no significant differences in gender and age between RM and no-RM patients. The demographics and clinical characteristics are provided in Table 1.

**TABLE 1:** Clinical information.

Parameters	RM (*n* = 42)	no-RM (*n* = 37)	*P*-value
Gender (M/F)	22/20	23/14	NS
Age (avg years)	66.79	61.97	NS
N0/N1/unknown	9/33/0	16/18/3	*
M0/M1/MX	16/0/26	23/2/12	*
T1/T2/T3/T4/unknown	2/3/36/1/0	3/7/24/1/2	NS
Stage I and Stage II/Stage III and Stage IV/unknown	40/2/0	34/2/2	NS

Tumor Node Metastasis classification (TNM): T stage refers to the situation of the primary tumor focus. With the increase of tumor volume, the depth of invasion and the range of adjacent tissue involvement, it is expressed by T1–T4 in turn. N stage refers to the regional lymph node involvement, which is represented by N0 when the lymph node is not involved. With the increase of the degree and scope of lymph node involvement, it is indicated by N1–N2 in turn. M stage means M refers to distant metastasis, with M0 for those without distant metastasis and M1 for those with distant metastasis; RM, recurrence or metastasis; no-RM, without recurrence and metastasis; NS, no significant differences. *Indicated *p*-value < 0.05.

### Bacterial profiles of pancreatic tissue differ between recurrence or metastasis and no-recurrence or metastasis patients

**FIGURE 2:**
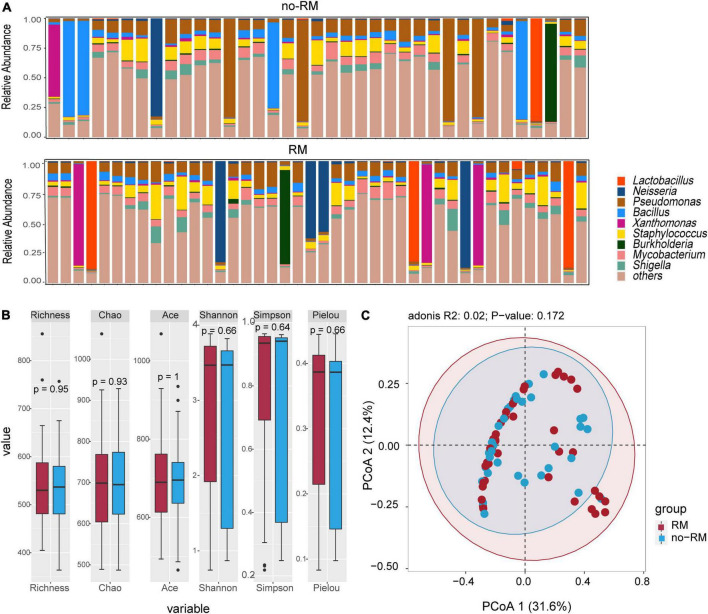
Difference in microbial composition and diversity between the two groups. **(A)** The top 10 genus levels in two groups; the stacked bar chart showed the composition of patient genus level in two groups of recurrence or metastasis. **(B)** Comparison of alpha-diversity of two groups based on different indexes. **(C)** Comparison of beta-diversity of two groups with PCoA. Wilcoxon test was used to detect variation between different groups based on the microbial composition at the genus level. Richness, Chao, and Ace index represent the richness of the microbial species; Shannon, Simpson, and Pielou index represent the diversity of the microbial species; RM, recurrence and metastasis; no-RM, no-recurrence and metastasis.

Previous microbial studies of pancreatic adenocarcioma have shown that bacterial composition shifted compared to non-diseased pancreatic (Pushalkar et al., 2018). Here, we intend to examine these compositional changes in distant metastatic PDAC. As shown in Figure 2, *Pseudomonas* dominated the tissue microbiome of pancreatic adenocarcioma with an average relative abundance of 12.8%, followed by *Staphylococcus* (7.3%) and *Bacillus* (6.9%) (Figure 2A). Further, there was no difference in alpha-diversity (richness, Chao, Shannon, and Sipmson) between RM and no-RM (Figure 2B). PCoA plot also showed no significant differences in bacterial communities between RM and no-RM (Bray–Curtis *P* = 0.172; Figure 2C). These data indicated similar global community alpha-diversity and beta-diversity between the RM and no-RM patients. Riquelme et al. (2019) found higher alpha-diversity in the tumor microbiome of long-term survival (LTS) patients compared with short-term survival (STS) patients. Our results demonstrate that metastasis in PDAC patients does not alter the overall tissue microbial community structure.

**FIGURE 3:**
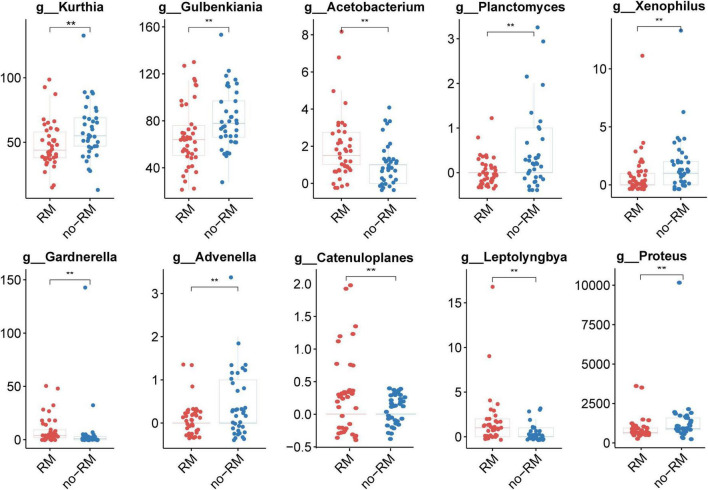
Ten potential biomarkers capable of distinguishing between RM and no-RM. Wilcoxon test was used to detect variation between different groups based on the relative abundance of tissue microbes, When the *p*-value was less than 0.01, 10 potential genus level microbial markers were identified; the boxplot was used to show the differences between the two groups; RM, recurrence or metastasis; no-RM, without recurrence and metastasis.

Next, we identified 10 potential biomarkers capable of distinguishing between RM and no-RM (Figure 3). The relative abundance of *Kurthia*, *Gulbenkiania*, *Acetobacterium*, *Planctomyces*, *Xenophilus*, *Gardnerella*, *Advenella*, *Catenuloplanes*, *Leptolyngbya*, and *Proteus* was significantly different between RM and no-RM (*P* < 0.01). Among them, the relative abundance of most bacterial biomarkers decreased in patients who developed recurrence or metastasis. Only the relative abundance of *Acetobacterium*, *Catenuloplanes*, and *Leptolyngbya* increased in the RM patients. The results demonstrated that decreased relative abundance of key bacteria in PDAC patient tissues may be a contributing factor to recurrence or metastasis. Although the overall microbial communities of RM and no-RM appear to be similar, recurrence and metastasis are still accompanied by increased or decreased relative abundance of some specific bacteria. These abundance-changing bacteria may be used as important indicators for clinical prediction of recurrence and metastasis of PDAC patients, so in-depth research such as experimental verification is urgently needed to reveal the underlying functional mechanisms of these bacteria.

### Transcriptome expression in pancreatic adenocarcioma patients carries information on recurrence or metastasis

**FIGURE 4:**
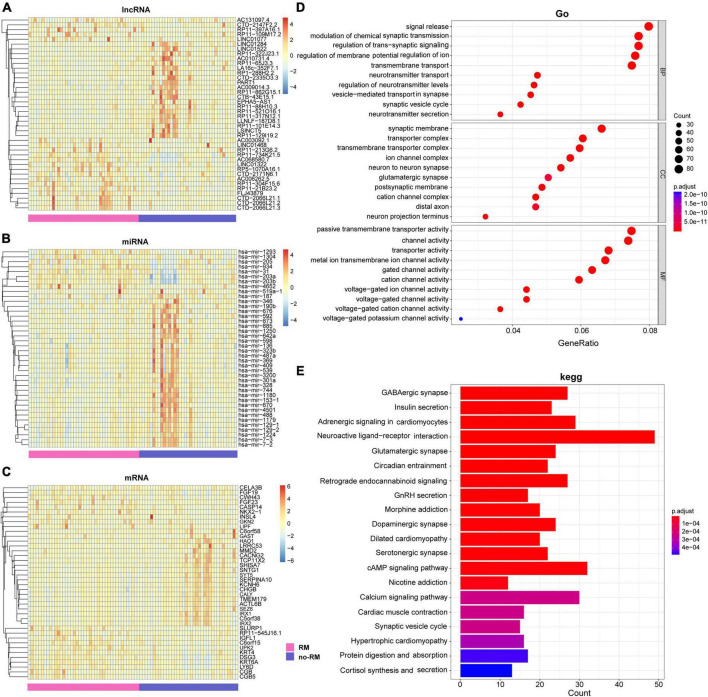
Difference analysis and enrichment analysis of different omics between two groups. The heat map of the DEGs of **(A)** lncRNA; **(B)** miRNA; **(C)** mRNA between the RM and no-RM group, the *x*-axis is the sample of two groups, and the *y*-axis is the top 40 expressions with significant differences screened by DEseq2. **(D)** GO analysis of DEGs between RM and no-RM. **(E)** KEGG analysis of the DEGs between RM and no-RM, the *X*-axis is the ratio of differentially expressed genes enriched in the corresponding pathway, and the *Y*-axis is the name of the pathway; BP, biological process category; CC, cellular component category; MF, molecular function category; RM, recurrence or metastasis; no-RM, without recurrence and metastasis.

Interactions and complex regulatory mechanisms among lncRNA, miRNA, and mRNA play key roles in the occurrence and development of multiple diseases (Huang, 2018; Liao et al., 2019; Cheng et al., 2020; Ma et al., 2020). In this study, instead of considering the regulation among lncRNA, miRNA, and mRNA, we analyzed the differences in these three transcriptomes between RM and no-RM separately. We performed a comprehensive analysis of the differential expression of each omics between the RM and no-RM. For lncRNA, 402 up-regulated and 288 down-regulated genes were identified. For miRNA, we identified 107 up-regulated and 44 down-regulated genes, while for mRNA, 3,074 up-regulated and 1,539 down-regulated genes were identified. After adjusting for the *P*-value, we obtained 309 significantly differentially expressed lncRNAs, 62 significantly differentially expressed miRNAs, and 1,287 significantly differentially expressed mRNAs (details in Supplementary Tables 1–3). Heatmap showed the differences in the expression levels of the top 40 lncRNAs, miRNAs, and mRNAs between RM and no-RM (Figures 4A–C).

Further, we explored the biological function of these significantly differentially genes (Figure 4D). For GO terms, all GO terms can be classified into three categories: (1) Biological process (BP), (2) Cellular component (CC), and (3) Molecular function (MF). First, for biological process, most of the BP terms have been confirmed to be related to the signal release and modulation of chemical synaptic transmission. Second, for cellular component, most of the CC terms can be clustered into synaptic membrane and transporter complex. Finally, as for molecular function, MF terms mostly contributed to the passive transmembrane transporter activity and channel activity. Furthermore, potential pathological pathways in PDAC metastasis were further analyzed with Kyoto Encyclopedia of Genes and Genomes (KEGG) annotations (Figure 4E). The results showed that the differentially expressed genes were mainly enriched in neuroactive ligand-receptor interaction, cAMP signaling pathway, and adrenergic signaling in cardiomyocytes, and other signaling pathways.

Chen et al. (2020) reported that for lower-grade glioma (LGG) and normal tissues, neuroactive ligand-receptor interaction was identified as differentially enriched pathway in KEGG. Also, in our study, a possible key pathway in RM patients with PDAC is neuroactive ligand-receptor interaction (Figure 4E). Different disease subjects share certain enriched pathways, which have also been reported in other studies (Priya et al., 2022). We strongly recommend further research on this topic to progressively improve the transcriptomic evidence on PDAC metastasis.

### Microbes are the best predictors of the recurrence and metastasis in patients with pancreatic adenocarcioma

**FIGURE 5:**
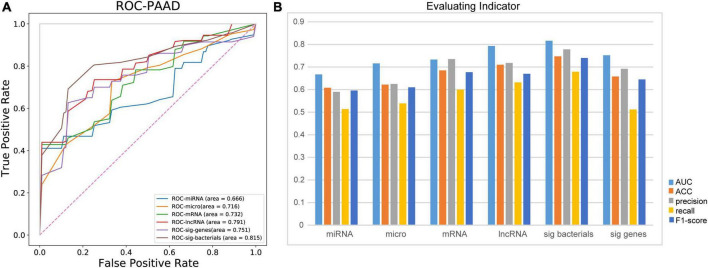
Ten identified bacterial biomarkers perform best in predicting recurrence and metastasis in patients with PDAC. **(A)** Comparison of AUC in patients with recurrence and metastasis predicted by different omics. **(B)** Evaluation of predictive ability of different evaluation indices for recurrence and metastasis of PDAC patients; micro, microbiome; sig bacteria, 10 identified bacterial biomarkers; sig genes, identified DEGs from mRNA data; AUC, area under curve; ACC, accuracy.

Predicting the recurrence or metastasis of pancreatic adenocarcioma patients plays a huge role in improving patient survival and reducing medical costs. Therefore, we further evaluated the performance of different omics in predicting the recurrence and metastasis in patients with pancreatic adenocarcioma (Figure 5A). Firstly, based on all the characteristics of each omics, RF fivefold cross-validation showed that lncRNA obtained the highest accuracy in predicting the recurrence and metastasis of pancreatic adenocarcioma patients (AUC = 0.791). However, when the 10 identified bacterial biomarkers were used as features, the prediction performance was the best with an AUC of 0.815. Besides AUC, other metrics (ACC, precision, recall, and F1-score) were also used to evaluate the predictive effect of each omics (Figure 5B). The results also showed that the 10 bacterial biomarkers performed best, which further indicate that the 10 bacteria may serve as potential biomarkers of recurrence and metastasis of PDAC.

### Recurrence and metastasis in pancreatic adenocarcioma patients are associated with reduced survival

Many studies have shown that microbes are closely related to the survival of cancer patients (Chattopadhyay et al., 2019; Peters et al., 2019; Riquelme et al., 2019). Besides, in this study, we found that the tissue microbiome significantly influenced the recurrence and metastasis of patients with PDAC. Therefore, we wonder whether recurrence and metastasis in patients are associated with survival and whether this association is driven by tissue microbes.

**FIGURE 6:**
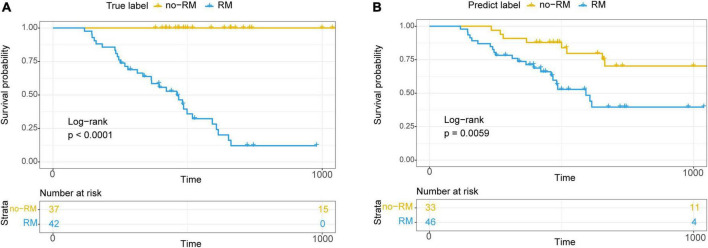
Kaplan–Meier survival curve showed significantly different overall survival between RM and no-RM. **(A)** Relationship between true recurrence and metastasis labels and overall survival of patients. **(B)** Relationship between recurrence and metastasis labels predicted by the model and the overall survival of patients; RM, recurrence or metastasis; no-RM, without recurrence and metastasis.

Based on these 10 bacterial biomarkers, all patients were classified to two clusters with machine learning classification model used previously. Then, survival analysis was conducted on the predicted clusters (Figure 6). First, survival time of RM patients were significantly shorter than those of no-RM patients (*P* < 0.0001; Figure 6A). Meanwhile, similar result was found when we conducted survival analysis on the two predicted clusters, that is, there was a significant difference in survival between the two clusters (*P* = 0.0059; Figure 6B). Our results demonstrate that the recurrence and metastasis in pancreatic patients are associated with reduced survival and this association is potentially driven by key tissue microbes.

## Discussion

The recurrence and metastasis have become a critical problem in cancer diagnosis, treatment, and metastasis (He et al., 2020; Shi et al., 2022). Through a comprehensive comparison of tissue microbes in non-metastatic and metastatic pancreatic adenocarcioma patients, we identified 10 bacteria that differentiate between RM and no-RM patients. Among them, the relative abundance of most bacterial biomarkers decreased in patients who developed recurrence or metastasis. Although there were significant differences in the expression patterns of multiple omics between RM and no-RM patients, the accuracy of these 10 bacteria in predicting recurrence and metastasis in pancreatic adenocarcioma patients was higher than that of other omics (lncRNA, miRNA, and mRNA). More importantly, these bacterial biomarkers potentially drive the association between metastasis and patient survival.

Beyond the simple description of tissue microbiome changes in pancreatic adenocarcioma patients, our study proposes the idea of microbe-based predictors for metastasis of PDAC. Groundbreaking, we identified 10 potential bacterial biomarkers. The microbe composition comparing normal esophagus with intestinal metaplasia, low grade dysplasia, high grade dysplasia, and adenocarcinoma showed significant decreases in the phylum Planctomycetes and the genus *Planctomyces* in diseased tissue compared with healthy controls and intrasample controls (Peter et al., 2020). We find that the relative abundance of *Planctomyces* in RM patients is significantly lower than that in no-RM patients. Bacterial dysbacteriosis, characterized by a predominance of *Gardnerella vaginalis* may accelerate the process of cervical carcinogenesis (Kovachev, 2020). Similarly, we also find an increased relative abundance of the genus *Gardnerella* in patients with recurrence or metastasis.

We find that the microbe-based predictor is more accurate compared with lncRNA, miRNA, and mRNA, possibly due to the tissue microbes play a dominant role in recurrence and metastasis of PDAC. The 10 bacterial biomarkers we identified could be used to clinically assist in the diagnosis of early stage pancreatic adenocarcioma patients for future recurrence and metastasis. Consequently, the medical costs and patient suffering will be greatly reduced. However, the detailed link between the tissue microbes and the pathological mechanism of pancreatic metastasis remains to be further clarified. It is of note that besides microbe and molecular biomarkers, histopathological images have been adopted to evaluate recurrence and metastasis risk for many cancers (Liu X. et al., 2022; Yang J. et al., 2022; Ye et al., 2022). Feasible directions to improve prediction accuracy include exploring more advanced machine learning models used in other related biological problems (Xu et al., 2020a; Meng et al., 2022) and integrating more types of prediction data.

The strength of our study includes two accurately divided pancreatic adenocarcioma cohorts with and without recurrence or metastasis within 1 year, the microbiome data at the site of initial cancer, and detailed follow-up information for all involved patients. Several limitations to the present study exist. First, the small sample size may make the findings less generalizable. Although we comprehensively compared the tissue microbiome of RM and no-RM patients, the absence of healthy controls is not conducive to underpinning the findings. In addition, we used the public data of TCGA database, which needs to be verified by the clinical data of the Chinese population. At the same time, the image information of patients was likely to be added to the framework of predicting recurrence and metastasis, and further model fusion will help to improve the prediction accuracy. Functional experiments are needed in the future to deeply explore the physiological mechanism of tissue microbes affecting the recurrence and metastasis of PDAC. Complete and organized experiments will help unravel pancreatic adenocarcioma metastases and aid clinicians in diagnosis.

## Conclusion

In conclusion, we characterize the system alterations of tissue microbiome in pancreatic adenocarcioma patients. We uncover the microbial signature associated with recurrence and metastasis of pancreatic adenocarcioma and develop a highly accurate microbe-based predictor for recurrence and metastasis diagnosis of PDAC.

## Data availability statement

Publicly available datasets were analyzed in this study. This data can be found here: https://portal.gdc.cancer.gov.

## Author contributions

HF contributed to conception and design of the study. SL and MY organized the database. SL, HF, and MY performed the statistical analysis. SL and LJ wrote the first draft of the manuscript. HF and LJ wrote sections of the manuscript. All authors contributed to manuscript revision, read, and approved the submitted version.

## Footnotes

1
https://portal.gdc.cancer.gov
